# Very ‘sticky’ proteins – not too sticky after all?

**DOI:** 10.1186/1478-811X-10-15

**Published:** 2012-06-14

**Authors:** Stephan M Feller, Marc Lewitzky

**Affiliations:** 1 Biological Systems Architecture Group, Weatherall Institute of Molecular Medicine, Department of Oncology, University of Oxford, Oxford, OX3 9DS, UK

## Abstract

A considerable number of soluble proteins in cells that biochemists try to analyze are difficult to handle because they seem to behave like sponges that ‘suck up’ many other proteins. We argue here that this behavior is commonly an artifact introduced by the experimenting scientist and that we need to study proteins like animals in the wild: they will only reveal many of their secrets when carefully observed in their largely undisturbed, natural environment. Computational studies that attempt to realistically model cellular protein networks must also factor in the diverse protein habitats to be found in cells.

## Commentary

Most protein biochemists and cell biologists know sticky proteins just too well. They are a pain to work with. They hang on to chromatography and antibody capture resins. When expressed in recombinant form, they form monstrously sized aggregates and bind to a plethora of irrelevant proteins from *E. coli* or other host cells. It seems a fair guess that thousands of scientific papers are fatally flawed by reporting supposedly specific but in reality entirely nonspecific interactions of VSPs.

In some cases, the stickiness is artificially inflicted by intentional protein modification, for example by the addition of a tag onto the protein in a bad spot, or by expressing inappropriate fragments that expose hydrophobic core regions. But even when great care is taken to avoid this, it appears that many proteins live their lives as molecular ‘glue balls’. How can they function in cells without disturbing the system? How can they not get permanently stuck when intracellular protein concentrations are often in excess of 200 mg/ml (a property that leads to an ‘extreme cuddling’ phenomenon known as macromolecular crowding)? How can they seemingly retain their stickiness for, in some cases, hundreds of million years of evolution?

The simple answer could be: many of them may not be so sticky after all when observed in their undisturbed natural habitat. We need to appreciate much more how different most experimental conditions that we routinely use are compared to the normal environment of proteins. In addition to a frequent lack of appropriate protein modifications on recombinant proteins, which, if present, could make proteins less sticky in vivo, possibly the greatest determinant in cells that prevents nonspecific stickiness is the intracellular compartmentalization of naturally occurring proteins in space and time.

We propose that we must forever say ‘Goodbye’ to the belief that most intracellular proteins float about their business like dumplings in a soup. This notion has been cherished by biochemists for multiple decades, but it has probably created a mental roadblock in many heads that may prevent those biochemists from taking into account new hypotheses which attempt to draw more holistic pictures of molecular protein actions in cells [1].

Most intracellular proteins probably act similar to ‘sophisticated’ human beings, who move about freely for short distances, but typically live in a defined village and use appropriate transport infrastructure when traveling to faraway places. They do not ever meet most of their fellow countrymen and interact preferentially with those they would like to meet, and they are usually protected from the environment when travelling on major traffic roads or highways.

The intracellular transport infrastructures, together with the signaling protein networks that steer virtually all biological processes, are key features of functional cell architectures of which we have only rudimentary knowledge so far.

We need to understand both, the molecular details of the individual protein building blocks AND the fundamental principles that shape cellular architectures to finally come a bit closer to grasping how cells really function. The newly emerging super-resolution imaging methods [2-8], if carefully applied, might help us on the way.

By contrast, these principles will probably never emerge from computational studies, if they treat proteins as mere dumplings in a cytoplasmic broth. If bioinformatics is to reach its full potential in cell biology, it must become a ‘bioinformatics of subcellular compartments’ [9,10]. It has to develop sophisticated methods to model appropriate sub-networks of signaling proteins in time and space and to interconnect these suitably so that they reflect the intricate and manifold architectural features that cells have developed during billions of years of evolution.
